# Clinical characteristics and cytokine profiles of adult obese asthma with type2 inflammation

**DOI:** 10.1038/s41598-023-41889-6

**Published:** 2023-09-08

**Authors:** Masako To, Yoshihito Arimoto, Natsue Honda, Yusuke Kurosawa, Kosuke Haruki, Yasuo To

**Affiliations:** 1 Department of Laboratory Medicine, Dokkyo Medical University, Saitama Medical Center, 2-1-50 Minami-Koshigaya, Koshigaya City, Saitama 343-8555 Japan; 2 Department of Allergy and Respiratory Medicine, The Fraternity Memorial Hospital, 2-1-11 Yokoami, Sumida, Tokyo, 130-8587 Japan; 3 Department of Pulmonary Medicine, International University of Health and Welfare Narita Hospital, 852 Hatakeda, Narita, Chiba 286-8520 Japan

**Keywords:** Metabolic disorders, Respiratory tract diseases, Epidemiology, Inflammation, Comorbidities

## Abstract

Obesity-related non-eosinophilic asthma has been identified as a phenotype of asthma. However, mepolizumab and omalizumab improve asthma control in severe asthma with obesity, implying that type-2 cytokines may be involved in the deterioration of control in obese asthma. Despite this, the clinical details of obese asthma with positive type-2 inflammation markers have not yet been reported. The objective of this study was to investigate the clinical characteristics of patients with obese asthma with positive type-2 inflammation markers. Adult obese asthmatic patients were enrolled and were classified into two groups: obese asthma with positive type-2 inflammation markers (T2) and obese asthma with negative type-2 inflammation markers (NT2), then data were compared. In total, 434 patients were enrolled (85% of patients were at GINA therapy step 4–5). The T2 group had a higher proportion of patients with persistent asthma since childhood and with allergic rhinitis. A higher percentage of patients used high-dose inhaled corticosteroids (ICS) and experienced acute exacerbations (annual exacerbation ratio ≥ 1) in the T2 group. Multivariate logistic regression analysis showed that the T2 group was independently associated with younger age, comorbidity of allergic rhinitis, persistent asthma since childhood, use of high-dose ICS, and acute exacerbation rate ≥ 1. Adipocytokine levels were similar between the groups. Collectively, obese asthma with positive type-2 inflammation markers is characterised by a higher percentage of persistent asthma since childhood and more severe asthma.

## Introduction

Obesity is considered one of the most severe health problems worldwide. Obesity affects a wide range of systemic diseases, including asthma. Obesity has been identified as a risk factor for poor asthma control^1–5^. Several mechanisms by which obesity affects asthma have been identified^6–8^. Clinical data have indicated a reduced response to inhaled corticosteroids in obese asthmatic patients^9–11^. Ex vivo findings have also shown that asthmatic patients who are overweight or obese show steroid resistance^12^. Adult onset-type obese asthma is characterized by elevated oxidative stress levels, female predominance, lower serum immunoglobulin E (IgE) levels, infrequent antigen-specific IgE antibody detection, and poor quality of life because of asthma symptoms^13–15^.

Considering the existing knowledge regarding the characteristics of obese asthma, it is widely considered that most adult asthmatic patients with obesity are characterised by non-type-2-inflammation. However, in clinical settings, several adult obese asthmatic patients have higher serum IgE levels and peripheral blood eosinophilia. Furthermore, sputum interleukin (IL)-5 levels in obese adult asthma were higher than in participants with normal weight^16^. In addition, both mepolizumab (a monoclonal antibody to IL-5)^17,18^ and omalizumab (a monoclonal antibody to human IgE)^19,20^ improved control of severe asthma with obesity.

Thus, there may be another phenotype in adult obese asthma in addition to the previously reported phenotype (adult obese asthma that is characterised by non-type 2 inflammation), that is, adult obese asthma with type-2-inflammation. However, the clinical and laboratory details of adult asthma with obesity with type 2 inflammation have not yet been reported. Specifically, to our knowledge, no clinical studies have directly compared the clinical features of patients with obese asthma with positive type-2 inflammation markers and those with negative type-2 inflammation markers. Therefore, this study aimed to investigate the clinical characteristics and serum cytokine profiles of patients with obese asthma with positive type-2 inflammation markers.

## Results

### Demographic characteristics of patients

In total, 434 obese asthmatic patients (351 in the T2 and 83 in the NT2 group) were enrolled in this study. The demographic characteristics and comorbidities are summarised in Table 1. The median age of the T2 group was significantly lower than that of the NT2 group (p < 0.001). The percentage of male patients was significantly lower in the NT2 group (p = 0.028). The percentage of patients with a family history of asthma (p = 0.005) was significantly higher in the T2 group. The percentage of patients with a comorbidity of allergic rhinitis was higher in the T2 group (p = 0.028). The percentage of patients with other comorbidities, except for dyslipidaemia, was similar between the groups.

**Table 1:** Demographic data of the patients.

	NT2	T2	p^§^
n	83	351	
Body mass index (kg/m^2^)	27.53 (26.22–30.30)	27.36 (26.03–29.54)	0.334
Age (year)	65 (51–74)	53 (44–66)	< 0.001
Sex: M	32 (39%)	185 (53%)	0.028
Smokers (Ex & Current)	23 (30%)	126 (40%)	0.148
Family history of asthma	23 (29%)	148 (46%)	0.005
Comorbidities			
Allergic rhinitis	46 (55%)	242 (69%)	0.028
Atopic dermatitis	5 (6%)	48 (13%)	0.062
Chronic sinusitis	7 (8%)	28 (8%)	0.826
GERD	17 (20%)	64 (18%)	0.640
Diabetes mellitus	17 (20%)	52 (15%)	0.242
Dyslipidaemia	27 (33%)	62 (18%)	0.004
Hypertension	22 (27%)	86 (25%)	0.778

Data are presented as the median (interquartile range) or n (%).

T2, patients with obese asthma with positive type-2 inflammation markers; NT2, patients with obese asthma with negative type-2 inflammation markers; GERD, gastroesophageal reflux disease.

^§^*p*-values for Mann–Whitney U tests or Fisher's exact tests.

### Characteristics of asthma and therapy

The disease characteristics and therapies for asthma are summarised in Table 2. The duration of asthma was longer in the T2 group (p < 0.001). A higher percentage of patients with a history of childhood asthma (p < 0.001) and persistent asthma since childhood (p < 0.001) was observed in the T2 group. Regarding asthma therapy, the percentage of patients using high-dose inhaled corticosteroids (ICS) was significantly higher in the T2 group (p = 0.001). In addition, the annual exacerbation ratio in the T2 group was significantly higher than that in the NT2 group (P = 0.004). The percentage of patients who suffered acute exacerbation (annual exacerbation ratio ≥ 1) was significantly higher in the T2 group (p = 0.005).

**Table 2:** Characteristics of asthma and its treatment.

	NT2	T2	p^§^
Duration of asthma	14 (4–19)	18 (8–32)	< 0.001
History of childhood asthma	5 (6%)	132 (38%)	< 0.001
Persistent asthma since childhood	3 (4%)	88 (26%)	< 0.001
Asthma therapy			
GINA therapy step 2 GINA therapy step 3 GINA therapy step 4 GINA therapy step 5	4 (5%) 14 (17%) 62 (74%) 3 (4%)	16 (5%) 27 (8%) 269 (76%) 39 (11%)	0.019
High-dose ICS	20 (24%)	197 (44%)	0.001
Daily use of OCS	3 (4%)	20 (6%)	0.591
Annual exacerbation ratio	0 (0–0)	0 (0–1)	0.004
Annual exacerbation ratio ≥ 1	14 (17%)	115 (33%)	0.005

Data are presented as the median (interquartile range) or n (%).

T2, patients with obese asthma with positive type-2 inflammation markers; NT2, patients with obese asthma with negative type-2 inflammation markers; GINA, Global Initiative for Asthma; ICS, inhaled corticosteroids; OCS, oral corticosteroids.

^§^*p*-values for Mann–Whitney U tests or Fisher’s exact tests.

### Pulmonary function tests and laboratory data

The results of pulmonary function tests and laboratory data are summarised in Table 3. FEV_1_/FVC (p = 0.024), %FEF_50_ (p = 0.005), and %FEF_75_ (p = 0.002) values in the T2 group were significantly lower than those in the NT2 group. As expected, the median eosinophil count (p < 0.001) and IgE level (p < 0.001) were significantly higher in the T2 group.

**Table 3:** Pulmonary function tests and laboratory data.

	NT2	T2	p^§^
FVC (L)	2.48 (1.96–3.07)	2.86 (2.26–3.65)	0.001
%FVC (%)	88 (78–94)	86 (76–97)	0.976
FEV_1_ (L)	2.09 (1.54–2.50)	2.24 (1.77–2.97)	0.018
%FEV_1_ (%)	89 (75–98)	84 (71–96)	0.080
FEV_1_/FVC (%)	84.3 (79.2–87.4)	80.1 (75.3–86.9)	0.024
%PEF (%)	82 (68–100)	84 (68–102)	0.602
%FEF_50_ (%)	82 (66–114)	70 (50–98)	0.005
%FEF_75_ (%)	67 (53–89)	56 (37–81)	0.002
Peripheral blood			
Eosinophils(/µL)	148 (96–198)	277 (159–468)	< 0.001
IgE (IU/mL)	20 (9–59)	227 (93–614)	< 0.001
RAST positive			
House dust	0 (0%)	242 (72%)	NA
Mite	0 (0%)	241 (72%)	NA
Cat	0 (0%)	57 (45%)	NA
Dog	0 (0%)	30 (34%)	NA
Candida	0 (0%)	14 (23%)	NA
Aspergillus	0 (0%)	21 (23%)	NA

Data are presented as the median (interquartile range) or n (%).

T2, patients with obese asthma with positive type-2 inflammation markers; NT2, patients with obese asthma with negative type-2 inflammation markers; FVC, forced vital capacity; FEV_1_, forced expiratory volume in 1 s; PEF, peak expiratory flow; FEF_50_, forced expiratory flow at 50% of the vital capacity; FEF_75_, forced expiratory flow at 75% of the vital capacity; IgE, immunoglobulin E; RAST, radioallergosorbent test; NA, not applicable.

^§^*p*-values for Mann–Whitney U tests or Fisher’s exact tests.

### Factors associated with adult obese asthma with positive type-2 inflammation

We investigated the factors independently associated with obese asthma with positive type-2 inflammation. We performed multivariable logistic regression analysis using the T2 group as the independent variable. Age, sex, duration of asthma, family history of asthma, persistent asthma since childhood, comorbidity of allergic rhinitis, comorbidity of atopic dermatitis, comorbidity of dyslipidaemia, %FEV_1_, %FEF_75_, GINA therapy step, use of high-dose ICS, and an acute exacerbation rate ≥ 1 were included as dependent variables in the analysis, with consideration of multicollinearity. A backward stepwise elimination method was used to construct the final multivariate model. The T2 group was independently associated with younger age, persistent asthma since childhood, comorbidity of allergic rhinitis, use of high-dose ICS, and acute exacerbation rate ≥ 1 (Table 4).

**Table 4:** Factors associated with adult obese asthma with positive type-2 inflammation markers.

	Adjusted OR (95% CI)	p
Age	0.979 (0.960–0.998)	0.030
Comorbidity of allergic rhinitis	1.919 (1.111–3.318)	0.020
Persistent asthma since childhood	5.075 (1.458–17.669)	0.011
High–dose ICS	2.479 (1.338–4.591)	0.004
Acute exacerbation rate ≥ 1	2.557 (1.292–5.142)	0.004

CI, confidence interval; ICS, inhaled corticosteroids; OR, odds ratio.

### Serum adipocytokine levels

**Figure 1:**
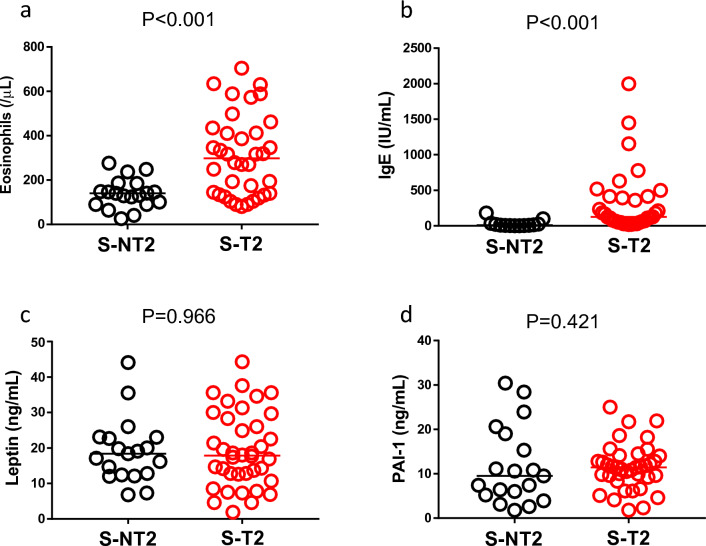
Type-2 inflammation markers and serum adipocytokine levels (**a**): peripheral blood eosinophil count, (**b**): IgE level, (**c**): leptin, (**d**): PAI-1 S-T2, patients with obese asthma with positive type-2 inflammation markers; S-NT2, patients with obese asthma with negative type-2 inflammation markers; PAI-1, plasminogen activator inhibitor-1.

Next, we compared serum adipocytokine levels between obese asthma with positive type-2 inflammation markers and those with negative type-2 inflammation markers. In this study, 57 obese patients with asthma were recruited (Table 5). As expected, serum eosinophil counts and IgE levels in the S-T2 group were significantly higher than those in the S-NT2 group (Fig. 1a and b). Serum leptin and PAI-1 levels did not differ between the S-T2 and S-NT2 groups (Fig. 1c and d).

**Table 5:** Demographic data of patients for serum mediator analysis.

	S-NT2	S-T2	p^§^
n	19	38	
Body mass index (kg/m^2^)	27.40 (25.30–28.40)	26.95 (25.63–28.71)	0.813
Age (year)	49 (44–65)	60 (47–75)	0.181
Sex: M	5 (26%)	15 (39%)	0.389
Smokers (Ex & Current)	2 (14%)	9 (26%)	0.475
Domestic exposure, Pet	6 (43%)	16 (44%)	1.000
Family history of asthma	4 (29%)	16 (46%)	0.344
Comorbidities			
Chronic sinusitis	1 (5%)	8 (21%)	0.247
GERD	6 (32%)	8 (21%)	0.516
Diabetes mellitus	3 (16%)	8 (21%)	0.735
Dyslipidaemia	5 (26%)	7 (18%)	0.509
Hypertension	6 (32%)	10 (26%)	0.758

Data are presented as the median (interquartile range) or n (%).

S-T2, patients with obese asthma with positive type-2 inflammation markers; S-NT2, patients with obese asthma with negative type-2 inflammation markers; GERD, gastroesophageal reflux disease.

^§^*p*-values for Mann–Whitney U tests or Fisher’s exact tests.

## Discussion

In this study, we analysed data on obese asthma (85% of patients were at GINA therapy step 4–5) and showed that a significant number of obese asthmatic patients with positive type-2 inflammation markers were detected in real-world data. One of the characteristics of the T2 group was that the percentage of adult patients with persistent asthma since childhood was higher. This finding suggests that a significant proportion of patients with obese asthma with positive type-2 inflammation markers are those who had early-onset type obesity-related asthma in childhood^13–15^. This early-onset type is characterised by higher serum IgE levels, the detection of antigen-specific IgE, and a more frequent history of asthma. In addition, The T2 group is also characterized by a higher proportion of high-dose ICS users and an independent association with an acute exacerbation rate of ≥ 1. Therefore, the T2 group is suggested to comprise a greater number of patients with severe asthma and a relatively higher proportion of patients with poor control.

An interesting finding of this study is the inflammatory marker profile of the participants. In this study, the adipocytokines (non-type-2 cytokines) in the serum were identical between individuals in the T2 and the NT2 groups. Of course, the type-2 inflammation markers were higher in patients in the T2 group. These findings indicate that patients with obese asthma with positive type-2 inflammation markers have both type-2 inflammation and adipocytokine-related non-type-2 inflammation while patients with obese asthma with negative type-2 inflammation markers mainly have adipocytokine-related non-type-2 inflammation. Therefore, overlapping inflammation in the patients with obese asthma with positive type-2 inflammation markers appears to be associated with more severe diseases in the phenotype. In fact, real-life data showed that the patients have more acute exacerbations (Table 4). The overlapping inflammation in patients with obese asthma with positive type-2 inflammation markers seems to cause a distinctive response to mepolizumab in obese asthma. Although mepolizumab is reported to be effective for obese asthma, the percentage of super-responders of the patients was reported to be low^21^. The mechanisms of increased type-2 inflammation in adult obese asthma seem heterogeneous. The relationship between asthma and obesity has been reported to be bidirectional^22^, which means that there is a possibility that obesity increases asthma severity and asthma increases the risk of obesity. Patients with severe asthma have a higher risk of obesity because of the frequent use of systemic corticosteroids and a sedentary lifestyle resulting from poor asthma control. In contrast, there is evidence suggesting that obesity-related factors might be involved in the upregulation of type-2 inflammation. Sputum eosinophil count, IL-5 expression, and CCR3 expression were positively correlated with body mass index (BMI)^23^ or visceral fat area^24^ in asthmatic participants. Additionally, an epidemiological study showed that eosinophil count was correlated with BMI in healthy participants^25^. Thus, there may be an association between obesity-related factors and increased type-2 inflammation.

The pulmonary function of patients with obese asthma with positive type-2 inflammation markers was lower than that of patients with obese asthma with negative type-2 inflammation markers in the univariable analysis. However, the variables were considered confounders by multivariable logistic regression analysis. The findings suggest that the patients may experience compromised lung function due to an increased frequency of exacerbations and/or prolonged asthma duration (persistent asthma since childhood), as presented in Table 4.

A key strength of this study was that all patients enrolled in the database were diagnosed with asthma by trained allergy specialists or physicians under training to be allergy specialists, based on the GINA guidelines. However, there are limitations to this study. A limitation is that only eosinophils and RAST scores were used to divide the enrolled patients into the T2 and NT2 groups. Using high-dose ICS or OCS would influence the levels of eosinophils in blood. Ideally, a method that also takes into account serum cytokines related to type-2 inflammation, sputum eosinophils, and fractional exhaled nitric oxide in the breath would be better. However, since there is no data available on these parameters in the database, it was realistic to divide the groups using only eosinophil counts and RAST scores. Another potential limitation is that the enrolled patients were exclusively Japanese individuals. However, we think the impact is minimal because the definition of obesity for Japanese individuals is used for this study. That is, the definition of obesity used was based on criteria established by the Japan Society for the Study of Obesity. This definition was specifically developed for Japanese individuals based on the observation that the threshold for the rise in obesity-related diseases in this population occurs at a BMI of 25 kg/m^2^ or higher. The World Health Organization's definition of obesity, on the other hand, is a BMI of 30 kg/m^2^ or higher, which is based on global surveys which indicate that the threshold for an increase in obesity-related diseases is a BMI of 30 kg/m^2^ or higher. Therefore, it is considered that the obese group in this study corresponds to the obese population worldwide in terms of physiological characteristics.

In conclusion, patients with obese asthma with positive type-2 inflammation markers are a distinct phenotype in obese asthma characterised by more persistent asthma since childhood and more severe asthma. Patients with this phenotype have both type-2 inflammation and obesity-related non-type-2 inflammation; the extent of the latter in this phenotype is as strong as that in patients with obese asthma with negative type-2 inflammation. The overlapping inflammation may cause more severe diseases in the phenotype. The mechanism of elevation of type-2 inflammation in obese asthma seems heterogeneous. Adult patients with obese asthma with positive type-2 inflammation markers are noteworthy; therefore, further study of this phenotype needs to be explored.

## Methods

### Study population

This study was based on the data obtained from ‘the FD asthma database’, which contains the clinical data of 1443 asthmatic patients (≥ 18 years old) who regularly visited the outpatient clinic of the Department of Allergy and Respiratory Medicine at The Fraternity Memorial Hospital in 2015. The database included demographic data, comorbidities, spirometry data (pre-bronchodilator values) under stable conditions of asthma, laboratory data under stable conditions of asthma, characteristics of asthma, types of controllers used under stable conditions, and the number of exacerbations. The duration of asthma was based on patient-reported data. The inclusion criteria for the database were (1) asthma diagnosed using the Global Initiative for Asthma (GINA) guidelines; (2) regular visits to our hospital and regular use of controller medication for asthma for ≥ 2 years before enrolment; (3) available spirometry data obtained under stable conditions; and (4) no other respiratory diseases potentially affecting the spirometry data, such as old tuberculosis, diffuse emphysema, bronchiolitis, interstitial lung disease, or diffuse lung diseases. Clinical and laboratory data were obtained from electronic medical records to establish the database.

Data of obese patients were extracted from the FD asthma database. We used the criteria for obesity for Japanese participants defined by the Japan Society for the Study of Obesity (BMI ≥ 25 kg/m^2^, criterion for obesity for Japanese people). The cut-off level was established because the optimal cut-off BMI for maximising the sensitivity and specificity for obesity-related disorders is 25 kg/m^2^ in Japanese people^26^.

### Study design

We classified the enrolled obese asthmatic patients into two groups: patients with obese asthma with positive type-2 inflammation markers (T2, peripheral blood eosinophil counts ≥ 300/µL or a specific allergen detected) and patients with obese asthma with negative type-2 inflammation markers (NT2, peripheral blood eosinophil counts < 300/µL, and no specific allergen detected), and data were compared between the groups. This study was approved by The Fraternity Memorial Hospital Research Ethics Committee and performed according to the Declaration of Helsinki.

### Data collection

All data were obtained from the FD asthma database. Severe asthma was defined based on the European Respiratory Society/American Thoracic Society (ERS/ATS) guidelines for severe asthma^27^. “Severe acute exacerbation of asthma” was defined as an event requiring an emergency room visit and hospitalisation for treatment with systemic corticosteroids or increased systemic corticosteroids from a stable maintenance dose for ≥ 3 days^28^. The annual exacerbation ratio was defined as the total number of severe acute exacerbations /total duration of the observed period (years).

### Collection and analysis of serum samples

Obese asthmatic patients were recruited from the Department of Allergy and Respiratory Medicine outpatient at Fraternity Memorial Hospital between April 2019 and December 2019 for this analysis. Patients who satisfied the following criteria were included: (1) had obesity; (2) had asthma diagnosed according to the Global Initiative for Asthma (GINA) guidelines; (3) had received regular medication for at least 1 year before enrolment; and (4) had no infection or asthma exacerbations requiring the administration of systemic corticosteroids within 4 weeks of enrolment. In addition, peripheral blood eosinophil counts and IgE levels under stable conditions of asthma were collected from the medical records.

Serum samples were collected from patients and stored at − 80 °C until analysis. Serum leptin and plasminogen activator inhibitor-1 (PAI-1) levels were measured using sandwich ELISA (R&D Systems, Minneapolis, MN). We classified the recruited obese asthmatic patients into two groups: patients with obese asthma with positive type-2 inflammation markers (S-T2, peripheral blood eosinophil counts ≥ 300/µL or a specific allergen detected) and patients with obese asthma with negative type-2 inflammation markers (S-NT2, peripheral blood eosinophil counts < 300/µL, and no specific allergen detected). Serum adipocytokines were compared between the groups. This study was approved by The Fraternity Memorial Hospital Research Ethics Committee and performed according to the Declaration of Helsinki. Written informed consent was obtained from all participating patients.

### Statistical analysis

Data were compared using the Mann–Whitney U test. Fisher’s exact test was used to compare the proportions of categorical variables. In addition, multivariable logistic regression analysis was performed to identify factors independently associated with the T2 group. Statistical significance was set at p < 0.05. All statistical analyses were performed using the SPSS ver. 28 software (IBM, Armonk, NY).

## Data Availability

The datasets generated during the current study are available from the corresponding author on reasonable request.

## Abbreviations

ATS: American Thoracic Society
BMI: Body mass index
ERS: European Respiratory Society
FEF_50_: Forced expiratory flow at 50% of vital capacity
FEF_75_: Forced expiratory flow at 75% of vital capacity
FEV_1_: Forced expiratory volume in 1 s
FVC: Forced vital capacity
GINA: Global Initiative for Asthma
ICS: Inhaled corticosteroids
IgE: Immunoglobulin E
IL: Interleukin
PAI-1: Plasminogen activator inhibitor-1
